# The Clinical Significance of the Primary Malignant Melanoma of the Lower Respiratory Tract and/or Lung Based on the Analysis of Published Case Reports and of Two Patients

**DOI:** 10.1155/DTE.1.169

**Published:** 1995

**Authors:** P. Barzó, K. Minik, P. Tuka, J. I. Kiss

**Affiliations:** 1 3rd Department of Pulmonology St. Ferenc Hospital Miskolc 3501 Hungary; 2 Department of Pathology County Hospital Miskolc 3526 Hungary; 3 partment of Pathology Semmelweis Hospital Miskolc 3501 Hungary; 4 Department of General and Thoracic Surgery Semmelweis Hospital Miskolc 3501 Hungary; 5 F.C.C.P., Departmental Head-Physician Specialist for Bronchopneumology and Internal Diseases III Department of Pulmonary-Internal Medicine Central Bronchology, St. Ferenc Hospital Miskolc 3501 Hungary

## Abstract

Considering the data and including two patients of the authors, there exist only 18 authentic cases of primary malignant melanoma of the lower respiratory tract and/or the lung. The tumor was localized in the endobronchial space in 7 cases and only once in the trachea. Endobronchial localization, together with the involvement of the surrounding lung tissue, was found in two cases, whereas in 8 patients, the tumor was found exclusively in the lung parenchyma. Successful resection could be performed in 14 of the 18 cases. Survival was influenced primarily by operability, and on actual size and extension. The authors question the role of the obduction in the diagnostic criteria, because most of the survivors, even up to ten years postsurgery were considered primary. Apart from the various imaging methods, diagnostic endoscopy (i.e., bronchoscopy) and the histology of the biopsy material are major aides in the diagnosis of primary character, location, and operability, as well as in the elaboration of the surgical plans, than it is usual in cases of other tumors.


*Diagnostic and Therapeutic Endoscopy*, 1995, Vol. 1, pp. 169–175
Reprints available directly from the publisher
Photocopying permitted by license only

© 1995 Harwood Academic Publishers GmbH
Printed in Malaysia

### The Clinical Significance of the Primary Malignant Melanoma of the Lower Respiratory Tract and/or Lung Based on the Analysis of Published Case Reports and of Two Patients

P. BARZÓ*, K. MINIK§, P. TUKA§§ and J. I. KISS§§

**3rd Department of Pulmonology, St. Ferenc Hospital, Miskolc 3501 Hungary*
*§Department of Pathology, County Hospital, Miskolc 3526 Hungary*
*§§Department of Pathology, Semmelweis Hospital, Miskolc 3501 Hungary*
*§§Department of General and Thoracic Surgery, Semmelweis Hospital, Miskolc 3501 Hungary*

*(Received January 6, 1994; in final form April 11, 1994)*

Considering the data and including two patients of the authors, there exist only 18 authentic cases of primary malignant melanoma of the lower respiratory tract and/or the lung. The tumor was localized in the endobronchial space in 7 cases and only once in the trachea. Endobronchial localization, together with the involvement of the surrounding lung tissue, was found in two cases, whereas in 8 patients, the tumor was found exclusively in the lung parenchyma. Successful resection could be performed in 14 of the 18 cases. Survival was influenced primarily by operability, and on actual size and extension. The authors question the role of the obduction in the diagnostic criteria, because most of the survivors, even up to ten years postsurgery were considered primary. Apart from the various imaging methods, diagnostic endoscopy (i.e., bronchoscopy) and the histology of the biopsy material are major aides in the diagnosis of primary character, location, and operability, as well as in the elaboration of the surgical plans, than it is usual in cases of other tumors.

**KEY WORDS:** melanoma malignum, primary melanoma in lung, primary melanoma in the bronchial tract

### INTRODUCTION

Eighty-eight percent of the primary malignant melanomas appear in the skin or the eye (1). As primary tumors, melanomas occur considerably less frequently in the oral cavity, upper respiratory tract, esophagus, liver, gall bladder, ovaries, cervix uteri, uterus, urogenital system, and leptomeninx. The malignant melanoma usually occurs as a metastatic tumor in the lower respiratory tract and/or lung (in 7% to 9% of those of cutaneous origin and in 2% of the autopsies) (2,3), and the primary forms of the same localization are extremely rare. It is assumed that the primary melanomas derive from the benign melanocytes moving together with the mesoderm and/or entoderm (4,5). Similar cells have been isolated from the mucosa of the esophagus and larynx. The latter organs derive from the 6th branchial arch as parts of the pulmonal system. Nevertheless, the presence of even benign melanocytes in the bronchopulmonary system is not usual (6). According to some suggestions, melanocytic metaplasy also may take place under various stimuli in epithelial cells, salivary glands, and nervous structures (7). It is possible also that some precursor cells (e.g., Kultschitsky cells) may undergo both neuroendocrine and melanocytic differentiation (8,9,15).

A careful analysis of the literature reveals that the majority of the really primary malignant melanomas located in the lower respiratory tract and/or lung have been described in English-speaking countries (Table 1); we find only Danish (11), French (12), and Spanish (13) publications in Europe, in addition to those in Great Britain (5,13a–21). Thus, we describe observations obtained from our two patients and survey the relatively few publications.

Address for correspondence: Dr. Pál Barzó, F.C.C.P., Departmental Head-Physician, Specialist for Bronchopneumology and Internal Diseases III, Department of Pulmonary-Internal Medicine, Central Bronchology, St. Ferenc Hospital, 3501 Miskolc, Hungary.

### CASE REPORTS

Mrs. B. J. is a 43-year-old patient. In her anamnesis there was a uterus exstirpation because of myomas. She recovered in our department on November 3, 1989, because of shadow seen by X-ray examination in the left thoracal region. She has been coughing for several weeks and is occasionally febrilic. Physical examination did not reveal any alterations on the skin; no lymph nodes could be found. The repeated posteroanterior summation X-ray examination and tomographic imaging of the left side in planes of sagittal direction revealed a round vascular shadow about 6 to 7 cm in the left hilar region (Figure 1). Bronchoscopy revealed the presence of a tumor at the beginning of the left upper lobe bronchus; the tumor was about 8 mm with a slightly uneven surface and brown-gray color (Figure 2). Histologic and cytologic analyses of the excised sample suggested that the alteration represented an anaplastic cancer of the lung consisting of intermediary cells. Because of this diagnosis, left pulmonectomy was performed on November 22, 1989.

Pathologic, histologic and macroscopic findings were left lung, 18 × 12 × 3 cm weighing 290 g. In one of the branches of the upper lobe bronchus we found a brown-gray tumor about 8 mm. The bronchial wall infiltrated by the tumor was in close proximity to a huge lymphnode of the hilus; this node was 5 × 4 × 3.5 cm, of tumorous appearance, part gray-white, part dark brown-gray, and soft and fragile.

Microscopic examination showed the tumor infiltrating all layers of the cartilagineous wall of the bronchus, and intruding into the bronchial lumen in polyplike form. The tumor surface is covered in part by typical ciliated respiratory epithelium, displaying in some places slightly atypical planocellular metaplasy or atypical melanocytic hyperplasy. Under the epithelium, several naevoid cell groups that were confluent with the tumor were observed.

**Table 1** Basic data of case reports recognizable as primary malignant melanoma of the lower respiratory tract and/or lung

| *Authors* | *No* | *Year* | *Age* | *Sex* | *Country* | *Survival time* | *Search for primary melanoma* |
|---|---|---|---|---|---|---|---|
| Salm | 1 | 1963 | 45 | M | England | 6 months | done |
| | | | Localization: left lower lobe bronchus, left upper lobe<br>Diagnosis from: bronchoscopic biopsy, surgical biopsy, autopsy<br>Operation: pneumonectomy | | | | |
| Reed and Kent | 2 | 1964 | 71 | M | USA | 10 years | done |
| | | | Localization: left lower lobe<br>Diagnosis from: surgical biopsy<br>Operation: lobectomy | | | | |
| Reid and Mehta | 3 | 1966 | 60 | F | New Zealand | 11 years | done |
| | | | Localization: right lower lobe bronchus<br>Diagnosis from: surgical biopsy<br>Operation: pneumonectomy | | | | |
| | 4 | | 35 | M | USA | postoperative death | done |
| | | | Localization: trachea lower third<br>Diagnosis from: surgical biopsy<br>Operation: trachearesection + plastica | | | | |
| Jensen and Egedorf | 5 | 1967 | 61 | F | Denmark | 7 months | done |
| | | | Localization: left upper lobe<br>Diagnosis from: surgical biopsy<br>Operation: segmental resection | | | | |
| Allen and Drash | 6 | 1968 | 40 | F | USA | not published | done |
| | | | Localization: right lower lobe<br>Diagnosis from: surgical biopsy<br>Operation: lobectomy | | | | |
| Taboada *et al.* | 7 | 1972 | 56 | M | USA | 1 year | done |
| | | | Localization: left lower lobe<br>Diagnosis from: surgical biopsy, autopsy<br>Operation: lobectomy | | | | |
| | 8 | 1972 | 40 | M | USA | 3 years | done |
| | | | Localization: left upper lobe<br>Diagnosis from: surgical biopsy<br>Operation: pneumonectomy | | | | |

**Table 1** Continued

| *Authors* | *No* | *Year* | *Age* | *Sex* | *Country* | *Survival time* | *Search for primary melanoma* |
|---|---|---|---|---|---|---|---|
| Walter *et al.* | 9 | 1972 | 33<br>Localization: left lung, pleura<br>Diagnosis from: thoractomical biopsy, autopsy<br>Operation: explorative thoracotomy | M | France | 22 months | done |
| Adebonojo *et al.* | 10 | 1979 | 55<br>Localization: right upper lobe bronchus<br>Diagnosis from: bronchoscopic biopsy, surgical biopsy<br>Operation: pneumonectomy | F | Nigeria | 3 years | done |
| Gephardt | 11 | 1981 | 47<br>Localization: right upper main bronchus<br>Diagnosis from: autopsy<br>Operation: no | M | USA | before interv. | done |
| Cagle *et al.* | 12 | 1983 | 80<br>Localization: the space between right central and lower lobes<br>Diagnosis from: thoracotomic biopsy, autopsy<br>Operation: explorative thoracotomy | M | USA | 5.5 months | done |
| Alghanem *et al.* | 13 | 1987 | 42<br>Localization: left lower lobe<br>Diagnosis from: surgical biopsy<br>Operation: lobectomy | F | USA | 2.5 years | done |
| Santos *et al.* | 14 | 1987 | 58<br>Localization: right lower lobe bronchus<br>Diagnosis from: surgical biopsy<br>Operation: lobectomy | M | Spain | 18 months | done |
| Bagwell *et al.* | 15 | 1989 | 62<br>Localization: left upper lobe bronchus<br>Diagnosis from: surgical biopsy<br>Operation: lobectomy | M | USA | 2 months | done |
| Jennings *et al.* | 16 | 1990 | 34<br>Localization: lingula bronchus<br>Diagnosis from: bronchoscopical biopsy<br>Operation: pneumonectomy | F | USA | 19 months | done |

Several small exulcerations also were found on the tumor surface. Most of the tumor mass consisted of spindle cells with hyperchromic nuclei; the cells were partly ordered in bundles of various directions or in vortexlike structures (Figure 3). Although the tumor was rich in cells, the occurrence of apparent mitoses was not high. The massive brown pigment depositions of the tumor cells proved to be melanin with silver stains; the distribution of this pigment is not uniform (Figure 4). The histologic structure of the lymph node is unrecognizable; it is filled completely with tumor tissue. Similar to other bronchial tumors in all aspects, the tumor is in part fusocellular in structure containing abundantly melanin pigment, and in part large, pale rounded or polygonal cellular in structure with large, vacuolated nuclei and mostly ballonized cytoplasm. These latter cells contain much less melanin. The HMB-45 melanoma marker procedure proved to be positive. The resection line of the left main bronchus did not contain tumorous tissue.

Diagnosis: Melanoma malignum bronchi lobi superioris pulmonis sinistri. Metastasis tumoris in lymphonodo hili pulmonis sinistri.

Opinion: The tumor is considered a primary bronchial melanoma. This opinion is supported by the following morphologic signs: a single tumor of central location in the bronchial lumen; the polypoid surface of the tumor covered by an epithelium displaying atypical melanocytic proliferation and atypical planocellular metaplasy, and epithelium naevoid cell nests confluent with the tumor (Figures 3–4).

The postoperative period was without complication. The patient received Deticen (dacarbasin) therapy in 12 cycles during the first postoperative year.

The metastatic origin of this tumor in the bronchus of the left upper lobe of lung could be excluded with high probability based on the negative results of dermatologic, otorhinolaryngologic, oculistic, and gynecologic, and abdominal ultrasound examinations. Because the skin did

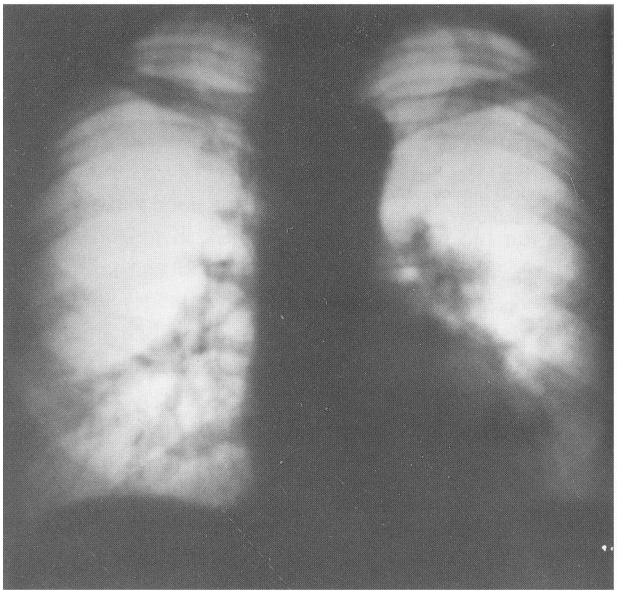

Figure 1 Chest X-ray of pa direction. In the left hilar region one can see a round shadow of about 6–8 cm.

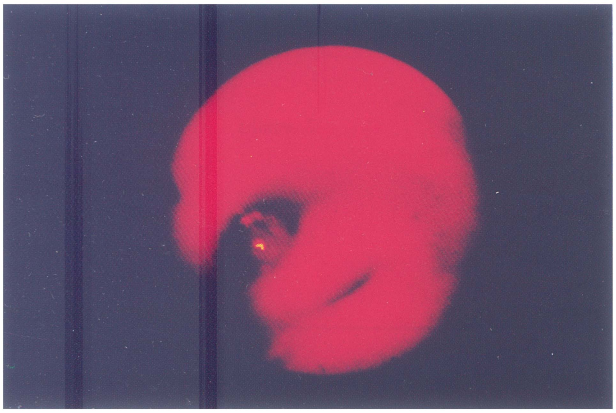

Figure 2 A tumor of about 6–8 mm can be seen in the lumen of the bronchus of the left upper lobe.

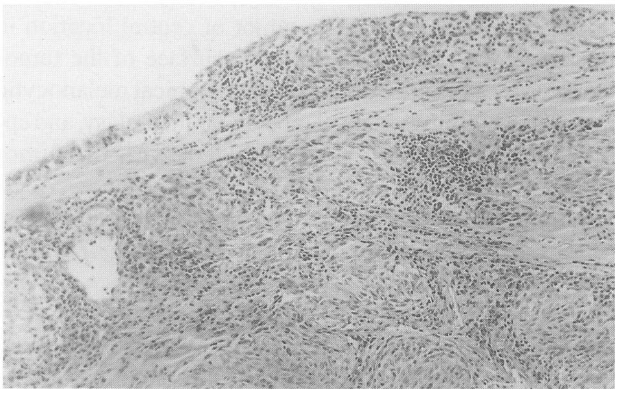

Figure 3 Spindle cells with hyperchromic nuclei, ordered partly in bundles, partly in vortexes × 100, hematoxylin-eosin staining.

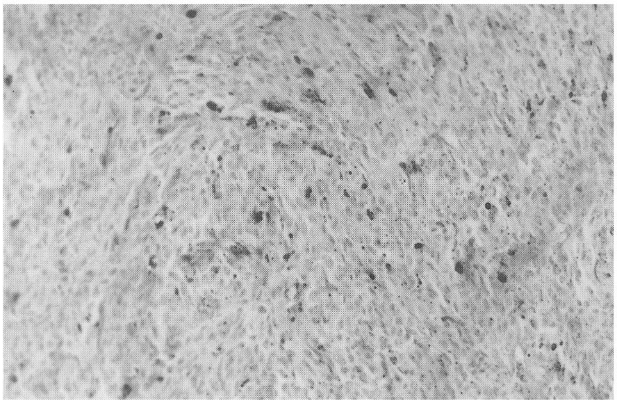

Figure 4 Massive brown pigment in the tumor cells with uneven distribution × 160, silver staining.

not show any alteration, excision from the skin was not performed. Currently, the patient suffers only from an effort dyspnoe and otherwise is without complaint. She gained about 10 kg body weight. Urine analyses using Thormälen and Jaksch tests for melanin proved negative.

Mrs. K. L., age 81 years complained of diffuse chest pains and dyspnoe. X-ray examination showed an egg-sized shadow in the segment 10 of the left side and a lymph node conglomerate in the hilus (Figure 5). Bronchoscopy was performed on November 21, 1990 and revealed that the lower part of the left main bronchus was almost completely closed by a tumorous gray-brown mass infiltrating the bronchial wall and penetrating from the direction of the bronchus of the lower lobe (Figure 6). To increase the bronchial lumen and improve the drainage, small parts of the tumor were removed. This intervention was repeated after 2 weeks, because the patient reported an improvement of her condition.

Histologically, the tumor is rich in cells and already has reached the surface of the bronchial mucosa. The tumor cells form solid groups, are medium-size with partly pale, eosinophilic cytoplasm, and contain numerous, focally localized, finely distributed, brownish, melaninlike pigment granules. The cell nuclei are hyperchromic and are ovoid or elongated, and some contain large nucleoli. There is an expressed cellular atypia and polymorphism. The brownish pigment tested positive for Grimelius and Masson-Fontana silver stainings, and the immunohistochemical HMB-45 melanoma result was also positive. All these results supported the diagnosis of melanoma malignum.

The biopsy material also was analyzed electromicroscopically; the cells containing the brownish melaninlike pigment also displayed numerous premelanosomes or melanosomes of high electron density (Figures 7 and 8).

The patient was observed for 4 months and was hospitalized for 23 days from this period. The analyses performed could not reveal the presence of malignant melanoma except in the bronchopulmonary location. The careful derma-

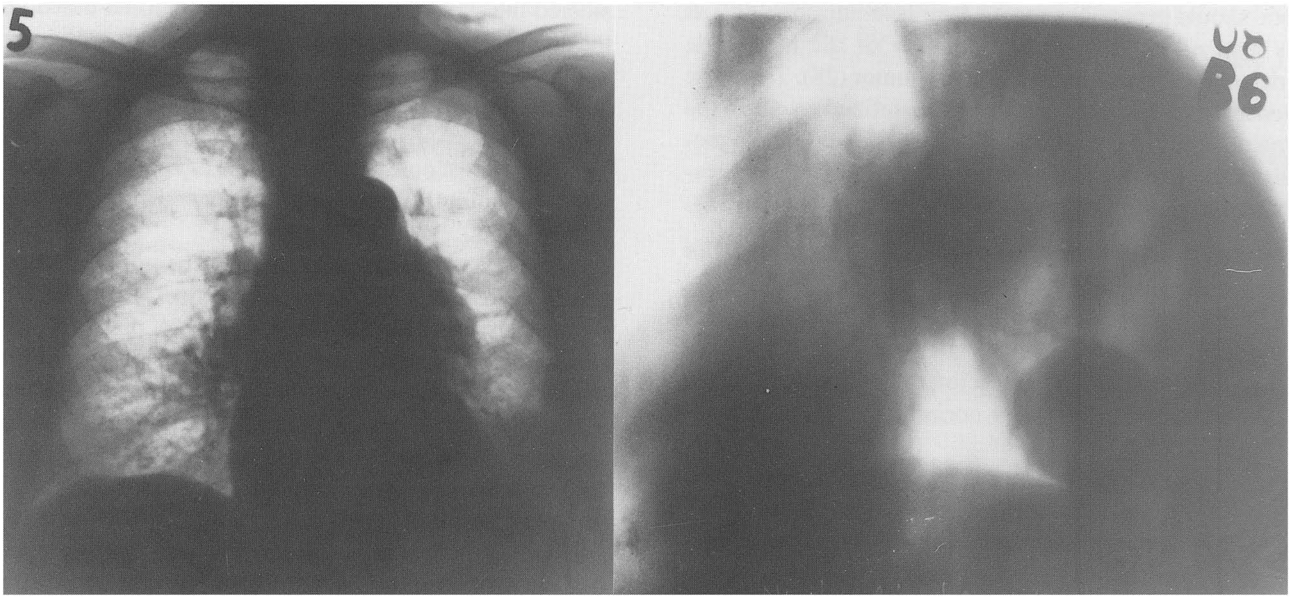

**Figure 5** Chest X-ray of pa and tomographic image of left lateral direction. An egg-sized shadow in the region of S-10 and lymphnode masses in the hilus.

tologic analyses remained negative for melanoma, therefore no skin biopsy was performed. Otorhinolaryngologic, oculistic, neurologic, and gynecologic, and abdominal ultrasound examinations were negative.

When the patient died from respiratory and circulatory insufficiency, autopsy could not be performed because family consent was not available.

### DISCUSSION

The first authentic primary malignant bronchopulmonal melanoma was described by Salm in 1963. In some of the cases described in later publications, one has to doubt the primary lung and/or bronchial origin. (22–26). The most serious issue is the exclusion of the metastatic origin of the tumor, because according to some opinions, skin melanomas may spontaneously disappear without any trace after they already have metastased. (2,27). However even if one accepts the possibility of the eventual, rare spontaneous regression of the primary skin melanoma,

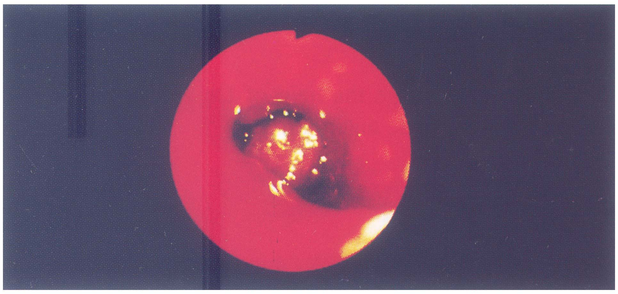

**Figure 6** A tumor considerably closing the lumen of the main left bronchus, deriving from the bronchus of the lower lobe.

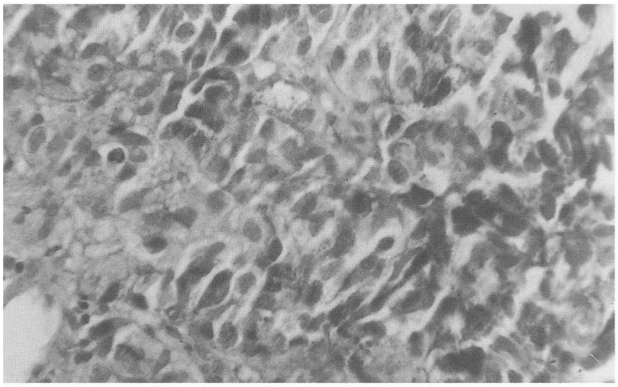

**Figure 7** Melanoblastic proliferative mass infiltrating the bronchial wall × 65, hematoxylin-eosin staining.

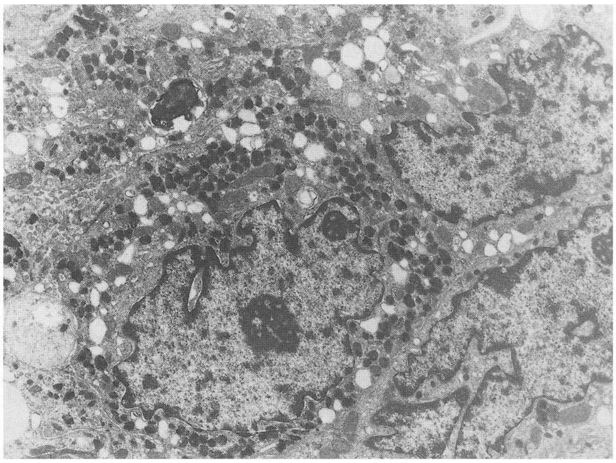

**Figure 8** Electron micrograph showing numerous premelanosomes and melanosomes of high electron density in the tumor cells. Primary magnification: × 4000.

one should generally expect a later generalization (27). It is also a fact that more than 15% of the metastatic melanomas have a hidden primary tumor (28). Therefore Jensen and Egedorf already have defined in 1967 the mainly clinical conditions necessary for the safe diagnosis of the primary malignant melanoma of the lower respiratory tract. These conditions include:

1. No previous removal of pigmented skin tumor or any kind of skin tumor
2. No removal of eye tumors, especially through enucleation
3. Surgically removed specimen with a solitary tumor
4. Morphology of tumor corresponds to the primary origin
5. No detectable melanoma in any other organ at time of surgical intervention
6. No primary malignant melanoma found, especially in the skin and the eyes, at autopsy.

The macroscopic and histologic properties of the tumor may offer further evidence for the origin of the tumor from the lower respiratory tract (6,15,21,29). Primary process is likely, if the tumor is polypoid and localized intraluminally, whereas the metastases are usually disseminated. The histologic conditions include:

1. Junctional alterations with the nestlike location or "dropping" of the melanoma cells
2. Invasion of melanoma cells in the bronchial epithelium at non-eroded places
3. Intraepithelial alterations that correspond unanimously to melanoma.

An accessory sign is considered to be the presence of naevuslike alteration in the bronchial epithelium nearby to the tumor and the melanoma flare that somewhat more distant from the tumor. (6,15,21). In our first reported case, the listed histologic criteria were present, whereas in our second case, not all criteria could be met, mainly because of the small size of the bioptic materials.

Generally speaking, the above criteria are considered valid, although the autopsy cannot always be performed, as described in some published cases, because of long postoperative survival or other reasons. On the other hand, autopsy itself has some limitations especially in answering the dilemma of primary tumor or metastasis. Therefore, if the obduction cannot be performed, the negative outcome of an extensive search for primary tumor in vivo can be accepted. Using modern endoscopic and imaging procedures, the probability of error can be reduced. Therefore, although the criteria defined by Jensen-Egedorf (1967) for the diagnosis of the primary malignant melanoma of the lower respiratory tract should be considered the standard, cases should be qualified based on the analysis of all test results and on common sense.

In both our cases, we found a solitary tumor with metastases in the hilus of the identical side. The malignant melanoma was proved by histologic, histochemic, and electron microscopic techniques. The tumors did not show any morphologic signs that suggested their metastatic origin.

According to the data (see Table 1), the primary malignant melanoma of the lower respiratory tract and/or lung involves both sexes almost equally. At time of diagnosis, the youngest patient was 33, the oldest 81 years of age. Of the 14 operated cases (lobectomy and pulmonectomy), two and one patients, respectively, are still alive, i.e., after 3 to 4 years, and one patient in each group survived after 10 to 11 years. Three patients were alive 2 to 30 months after the intervention. Five patients died between 5.5 and 22 months, and one patient died during the postoperative phase of tracheal resection. There was one patient who underwent successful right lower lobectomy the whose survival is not known.

The tumor was endobronchial in seven patients, and was intratracheal in one patient. The endobronchial location was accompanied by the involvement of the adjacent lung area in two persons, and exclusive parenchymal location was seen in eight cases. These facts indicate the significance of the bronchoscopy in the diagnosis. Operability and Survival are determined not by the bronchial or parenchymal location but by extension and circumscription of the tumor. The presence of a solitary, metastatic hilar lymph node, even if it is of considerable size, does not predict an unequivocal bad prognosis, as was shown in our first case.

In the therapy of the primary malignant melanoma of the lower respiratory tract, surgical removal is most important; considerable survival can be expected only from this intervention. The cytostatic treatments applied so far in the inoperable cases did not give any result; the effects of the adjuvant treatments cannot be measured either. Therefore, the main task is to detect the tumor as early as possible and if operable, surgical intervention must be performed.

### ACKNOWLEDGMENTS

Authors acknowledge the help of Dr. P. Degrell for the electron microscopic analyses.

### REFERENCES

1. Dorn HR, Cutler SJ. Morbidity from cancer in the United States. *Public Health Monograph* 1959. No. 56. Washington, D.C., U.S. Gov. Printing Office. cit. Jensen O. A, Egedorf J.: Scand J Resp Dis, 1967;48:127–135

2. Dasgupta TK, Brasfield RD, et al. Primary melanomas in unusual sites. *Surg Gynecol Obstet*, 1969;128:841–848
3. Gromet MA, Ominsky SH, et al. The thorax as the initial site for systemic relapse in malignant melanoma: a prospective survey of 324 patients. Cancer, 1979;44:776–784
4. Goldmann JL, Lawson W, et al. The presence of melanocytes in the human larynx. Laryngoscope, 1972;82:824–835
5. Taboada CF, McMurray JD, et al. Primary melanoma of the lung. Chest, 1972;62:629–631
6. Jennings IA, Axiotis CA, et al. Primary malignant melanoma of the lower respiratory tract. Am J Clin Pathol, 1990;94:649–655
7. Shivas AA, Mactennan WD. "Melanogenic metaplasia" of mucous glands. Br J Cancer, 1963;17:411–414
8. Cebelin MS. Melanocytic bronchial carcinoid tumor. Cancer, 1980;46:1843–1848
9. Gould VE, Memoli VA, et al. Neuroendocrine carcinomas with multiple immunoreactive peptides and melanin production. Ultrastruct Pathol, 1981;2:199–217
10. Grazer R, Cohen SM, et al. Melanin-containing peripheral carcinoid of the lung. Am J Surg Pathol, 1982;6:73–78
11. Jensen OA, Egedorf J. Primary malignant melanoma of the lung. Scand J Resp Dis, 1967;48:127–135
12. Walter P, Fernandes C, et al. Mélanome malin primitif pulmonaire. Ann Anat Pathol, 1972;17:91–99
13. Santos F, Entrenas IM, et al. Primary bronchopulmonary melanoma. Scand J Thorac Cardiovasc Surg, 1987;21:187–189
13a. Adebonojo SA, Durodola JI. Primary malignant melanoma of the bronchus. J Nat Med Assoc, 1979;71:579–881
14. Alghanem AA, Mehan J, et al. Primary malignant melanoma of the lung. J Surg Oncol, 1987;34:109–112
15. Allen MS, Drash EC. Primary melanoma of the lung. Cancer, 1968;21:154–159
16. Bagwell SP, Flynn SD, et al. Primary malignant melanoma of the lung. Am Rev Respir Dis, 1989;139:1543–1547
17. Hsu CW, Wu SC, et al. Melanoma of lung. Chin Med J, 1962;81:263–266
17. Cagle P, Mace ML, et al. Pulmonary melanoma primary vs. metastatic. Chest, 1984;85:125–126
18. Gephardt GN. Malignant melanoma of the bronchus. Human Pathol, 1981;12:671–673
19. Reed RJ, Kent EM. Solitary pulmonary melanomas: two case reports. J Thorac Cardiovasc Surg, 1964;48:226–231
20. Reid JD, Mehta VT. Melanoma of the lower respiratory tract. Cancer, 1966;19:627–631
21. Salm R. A primary malignant melanoma of the bronchus. J Path Bact, 1963;85:121–126
22. Carlucci GA, Schleussner RC. Primary melanoma of the lung. J Thorac Cardiovasc Surg, 1942;11:643–649
23. Carstens PHB, Kuhus JG, et al. Primary malignant melanomas of the lung and adrenal. Human Pathol, 1984;15:910–914
24. Robertson AJ, Sinclair DJM, et al. Primary melanocarcinoma of the lower respiratory tract. Thorax, 1980;35:158–159
25. Rosenberg LM, Polanco GB, et al. Multiple tracheobronchial melanomas with ten-year survival. JAMA, 1965;192:133–135
26. Weshler Z, Sulkes A, et al. Bronchial malignant melanoma. J Surg Oncol, 1980;15:243–248
27. Smith JL, Stehlin JS, Spontaneous regression of primary malignant melanomas with regional metastases. Cancer, 1965;18:1399–1415
28. Kopf AW, Bart RS, et al. Malignant melanoma: a review. J Dermatol Surg Oncol, 1977;3:41–125
29. Dail DJ, Hammar SP. *Pulmonary Pathology*. Springer-Verlag. New York,1988

